# The Incidence of Obstructive Sleep Apnea in Patients with Dento-Skeletal Malformation

**DOI:** 10.3390/dj12070225

**Published:** 2024-07-19

**Authors:** Giuseppe D’Amato, Mattia Todaro, Gianmarco Saponaro, Paolo De Angelis, Alessandro Moro, Francesca Azzuni, Benedetta Capasso, Giulio Gasparini

**Affiliations:** 1 Faculty of Medicine and Surgery, Unicamillus International Medical University, 00131 Rome, Italy; dottgdamato@gmail.com; 2 Maxillo-Facial Unit, Policlinico A. Gemelli, Università Cattolica del Sacro Cuore, Largo Agostino Gemelli, 8, 00168 Rome, Italy; alessandro.moro@unicatt.it (A.M.); francesca.azzuni01@icatt.it (F.A.); benedettacapasso01@gmail.com (B.C.); 3 Department of Head and Neck and Sensory Organs, Division of Oral Surgery and Implantology, Fondazione Policlinico Universitario A. Gemelli IRCCS-Università Cattolica del Sacro Cuore, 00168 Rome, Italy; dr.paolodeangelis@gmail.com; 4 Department of Head and Neck and Sensory Organs, Division of Pre Prosthetic Surgery, Fondazione Policlinico Universitario A. Gemelli IRCCS-Università Cattolica del Sacro Cuore, 00168 Rome, Italy; giulio.gasparini@unicatt.it

**Keywords:** obstructive sleep apnea, polysomnography, orthognatic surgery, dento-skeletal malformation, maxillary, mandible

## Abstract

**Purpose:** The aim of this article is to analyze the incidence of undiagnosed obstructive sleep apnea (OSA) in patients affected by dento-skeletal malformation. We also evaluated the patterns most affected by the condition and calculated the post surgical changes. **Methods:** We conducted a retrospective cohort study on 71 patients including 35 men and 36 women. The patients studied were affected by dento-skeletal class II and III malformations and underwent bimaxillary orthognathic surgery in all cases. Patients were evaluated with polysomnography before surgery and at least 6 months after surgery to assess any improvement or worsening of the apnea hypopnea index (AHI) index. Regarding AHI evaluation criteria, an AHI > 5 was considered indicative of OSA, 4 < AHI < 5 was considered borderline and AHI < 4 was considered indicative of non-OSA. We also considered demographic variables like age at the time of intervention and gender, and anatomical variables like the pattern of the dento-skeletal deformity and the presence or absence of maxillary hypoplasia. Qualitative variables were described as absolute and relative frequencies, while quantitative variables were summarized as mean and standard deviation. To quantitatively express the relationship between two variables, the correlation coefficient was calculated. The covariance array was used to evaluate multiple correlations. **Results:** Our study shows that there is a significant percentage (33%) of patients who undergo orthognathic surgery with an AHI > 5 and also a percentage of patients (11%) who can be considered to be “borderline.” It emerges that the pattern most at risk is the one characterized by retruded maxilla and patients with dento-skeletal class II. Considering the post surgical period, the statistical analysis shows that after surgery, only 8% of malformed patients present an AHI > 5, compared to the 20.5% described in the Italian population. **Conclusions:** In patients who receive orthognathic surgery, the presence of obstructive sleep apnea is significantly higher than in the general population. When planning the surgical correction of a dento-skeletal malformation, the surgeon must aim not only for the esthetics results, but also for proper stomatognathic and respiratory function; this cannot be achieved without taking polysomnography information into account.

## 1. Introduction

Obstructive sleep apnea (OSA) syndrome is a pathological condition characterized by recurrent episodes of the collapse of the upper airways, leading to a reduction in blood oxygen levels and usually culminating in a transient awakening episode; it is a major risk factor for the development of cardiovascular and metabolic diseases resulting in a reduced life expectancy and significant economic and social implications [1]

The symptomatology that most frequently describes this syndrome is both nocturnal and diurnal: snoring, the witnessed apneas and daytime drowsiness are the pivotal symptoms. The crucial instrumental examination for studying sleep disorders is polysomnography that documents the pattern and changes in a few physiological parameters during different stages of sleep. The pathogenesis of OSAS is related to several factors, some of which are in turn associated with dento-skeletal malformations, conditions caused by non-harmonic developments of the upper jaw and/or mandible that lead a narrowing of the upper airways and that expose a person to the risk of developing this syndrome [2]. In particular, the condition that is reported to be more frequently related to OSA is maxillomandibular retrusion, which seems to be related to a reduced upper airway volume caused from this anatomical condition [3].

It is estimated that nearly 1 billion adults between the ages of 30 and 69 worldwide are affected by obstructive sleep apnea. In Italy, according to Benjafield’s study, 20.5% of the population between 30 and 69 years old has an AHI (Apnoea-Hypopnoea index) of five events per hour and 12% has an AHI of fifteen or more events per hour. Apnea was characterized by the absence of airflow for more than 10 s. Hypopneas were defined as any reduction in airflow exceeding 50%, lasting longer than 10 s, and leading to either arousal or oxyhemoglobin desaturation. The presence of OSAS is not always correlated to the presence of symptoms of which the patient is aware, which is why it is an under-diagnosed and under-treated condition. Many studies show that patients affected by a dento-skeletal malocclusion are at a major risk of suffering from OSAS or from a chronic sleep-breathing disorder (SBD) [4].

Ristow et al. affirmed that the patient’s facial profile is a pivotal element in determining the risk of OSAS and that many patients with OSA present maxillary or mandibular hypoplasia [5].

Most of the studies that describe the connection between OSAS and dento-skeletal malformations refer to patients affected by severe OSA cases (AHI < 5) [6]. Less is known about the incidence of silent OSAS in patients affected by dento-skeletal malformations who are candidates for a surgical correction.

Posnick’s study found “silent” obstructive sleep apnea to be frequent in patients with maxilla-mandibular malformations; the prevalence in subjects with retrusive jaw patterns surpassed the estimated prevalence of OSA in the general population [7].

The purpose of this observational retrospective study was to evaluate the incidence of silent obstructive sleep apnea in patients with dento-skeletal malformations who undergo orthognathic surgery and the potential change in AHI pre- and postoperatively. 

Orthognathic surgery is designed to reposition the jaw, mandible, and chin with the purpose of enhancing facial balance and proportions and it is known that skeletal base movements can have a significant impact on the upper airway volume, which is highly influenced by anatomical structures; therefore, the study of the airway becomes essential in successful surgical planning.

## 2. Materials and Methods

A retrospective cohort study was designed. The study considered 71 patients treated for dento-skeletal malformation by the Complex Operating Unit of Maxillofacial Surgery at the IRCCS Policlinico Universitario Agostino Gemelli Foundation in Rome between 1 January 2020 and 31 December 2022.

The inclusion criteria included the following:

- Patients affected by a dento-skeletal malformation;
- Patients who are candidates to orthognathic surgery;
- Patients older than 18 years of age;

And the exclusion criteria included the following:

- Anamnesis of respiratory system diseases;
- Diagnosis of OSA prior to the first maxillofacial assessment;
- Patients with previously treated dento-skeletal malformations related to genetic syndromes, post-traumatic disorders or neoplastic diseases.
- Patients with BMI variation between the pre- and postoperative phases.

All patients underwent LeFort I osteotomy, bilateral mandibular sagittal osteotomy, and possibly associated genioplasty surgeries. The surgeries were combined in various ways depending on the surgical schedule.

All patients underwent Epworth Sleepness Scale (ESS) testing and polysomnography evaluating the AHI (Apnea-Hypopnea Index) and RDI (Respiratory Distress Index) indices. BMI (Body Mass Index) was also calculated.

At least 6 months after surgery, patients underwent polysomnography again to assess any improvement or worsening of the AHI.

General parameters such as sex, age, and general medical history were collected. A standard medical checkup was then performed.

Each patient was given an identification code so that the data could be processed anonymously.

The results of the study visit were entered into a database (Microsoft Excel—Microsoft 365) so that statistical analysis could then be carried out.

Predictive variables were chosen for our study and were grouped into the following categories:

- Demographic variables
  - Age at the time of intervention.
  - Gender.
- Anatomical variables
  - Pattern of dento-skeletal deformity.
  - Presence or absence of maxillary hypoplasia.
- Medical variables
  - BMI.
  - Apnea-Hypopnea Index (AHI) before orthognathic surgery.
  - Apnea-Hypopnea Index (AHI) post orthognathic surgery.
  We used the following AHI evaluation criteria:
  - An AHI > 5 equates to a patient with OSA.
  - An 4 < AHI < 5 equates to a patient with borderline OSA.
  - An AHI < 4 equates to a patient without OSA.
- Outcome variables

The prevalence of undiagnosed OSAS in subjects evaluated for dento-skeletal malformations who received orthognathic surgery in our department between 1 January 2020 and 31 December 2022.

In the discussion, the prevalence of OSAS in subjects undergoing orthognathic surgery was also compared with that of the general population.

Our analysis refers to two specific periods, the pre- and postoperative periods.

In the pre-surgery period, the following were studied:
  - The relationship between the AHI of the general cohort and that of the general population.
  - The relationship between an AHI > 5 and malformations, for both DSC IIs and DSC IIIs.
  - The relationship between an AHI > 5 and malformations, in DSC III with maxillary hypoplasia, both compared to DSC III and to the general cohort.
  - The relationship between an 4 < AHI < 5 and malformations, for both DSC II and DSC III.
  - The relationship between an AHI 4 < AHI < 5 and malformations, in DSC III with maxillary hypoplasia, both compared with DSC III and with the cohort overall.
In the post-surgery period, the following were studied:
  - The relationship between the AHI in a general cohort and in the general population.
  - The relationship between an AHI > 5 and malformations, both for DSC II and DSC III.
  - The relationship between an AHI > 5 and malformations, in DSC III with maxillary hypoplasia, both compared to DSC III and to the general cohort.
  - The relationship between an 4 < AHI < 5 and malformations, for both DSC II and DSC III.
  - The relationship between an 4 < AHI < 5 and malformations, in DSC III with maxillary hypoplasia, both compared with DSC III and compared with the cohort in general.

After that, in order to express the presence and any intensity of the link between two variables quantitatively, the correlation index was calculated. 

The variables considered included the following:

- Patients with an AHI > 5 and dento-skeletal class II or III malformations.
- Patients with an AHI < 5 but >4 (borderline) and dento-skeletal class II or III malformations.

The correlation array, also called the covariance array, was then used to calculate the multiple correlations between:

- DSC II, BMI, age and AHI;
- DSC III, BMI, age and AHI.

Moreover, the presence of any difference between pre- and post-surgery AHI was examined and the percentage of patients whose AHI value worsened post orthognathic surgery was calculated.

Linear plots were constructed based on the data that emerged.

## 3. Results

In total, we considered 71 patients including 35 men and 36 women ranging in age from 18 to 55 years with a mean age of 26.2 years. Twenty-one patients (29.5%) had class II dento-skeletal malformations according to Angle. Fifity (70.5%) patients had class III dento-skeletal malformation according to Angle. Of these, six (8.4% of the general cohort and 12% of patients with DSC III) had maxillary hypoplasia calculated based on cephalometric criteria [8].

### 3.1. Pre Surgery

We calculated the number of patients with malformations examined who had an AHI > 5 and a percentage of 33% resulted. 

In particular, 16 DSC II (76%) patients had an AHI > 5 and 8 patients with a DSC III malformation (16%) had an AHI > 5.

Of the patients with a DSC III malformation, we also highlighted the possible presence of maxillary hypoplasia and calculated the percentage of patients with an AHI > 5 falling into this category compared with the total number of patients with a DSC III malformation in the study and compared with the total number of patients with malformations considered in the study. 

It was found that 62% of patients with a DSC III malformation and an AHI > 5 were affected by maxillary hypoplasia. Next, calculating the ratio of patients with maxillary hypoplasia to those of the general cohort with an AHI > 5 showed that 25% had maxillary hypoplasia.

We also considered an AHI that was not strictly pathological but at the borderline of <5 but >4 and it turned out that of 21 patients with a DSC II malformation, 3 had an 4 < AHI < 5, so 14%, while out of 50 patients with a DSC III malformation, 5 had an 4 < AHI < 5 so 10%.

Among the patients with a DSC III malformation and a borderline AHI, only one out of five also had maxillary hypoplasia, and compared to the total malformed patients taken into the study with a borderline AHI, it was found that 12.5% of the patients also had maxillary hypoplasia.

### 3.2. Post Surgery

Of the malformed patients, 8% examined had an AHI > 5. 

Out of 21 patients with a DSC II malformation, only one presented with an AHI > 5 in the postoperative period, thus representing a percentage of 5%.

Out of 50 patients with a DSC III malformation, five had an AHI > 5 in the postoperative period, representing a percentage of 10%.

Considering the patients with a DSC III malformation and an AHI > 5 in the postoperative period, 80% of the patients had maxillary hypoplasia (four out of five patients), while considering the total number of patients examined with malformations and with an AHI > 5 in the postoperative period, the percentage was 66% (four out of six patients).

Out of 21 patients with a DSC II malformation, two had a borderline postoperative AHI, equating to 9.5% of patients.

Among 50 patients with a DSC III malformation, four had a borderline postoperative AHI, equating to 8% of patients.

Among the patients with a DSC III malformation and a borderline postoperative AHI, two out of four also had maxillary hypoplasia, equating to 50% of the patients, while compared to the total number of patients examined with malformations and a borderline postoperative AHI, it was found that two out of six patients also had maxillary hypoplasia, equating to 33% of the patients.

Considering the postoperative AHI also showed that 1 of 71 patients examined had worsened: his original AHI was 4.8 and after orthognathic surgery, it increased to 6.3, so it increased by about 3%.

This patient belongs to the DSC III group and has maxillary hypoplasia.

Calculating the correlation between an AHI > 5 and dento-skeletal malformation, considering DSC II and III, a significant correlation coefficient of −0.58 results. This means that there is an inverse correlation where an AHI > 5 is correlated more with a DSC II than with a DSC III malformation. 

In contrast, the correlation calculated between an AHI < 5 but >4 and the dento-skeletal malformation classes was −0.06, so it was not significant. In terms of the covariance matrix, the only significant value emerges in the correlation between BMI and an AHI > 5 in patients with a DSC II malformation. No kind of significant correlation value was found in patients with a DSC III malformation.

Finally, estimating the regression model relating the variable AHI with the covariates, sex, age, BMI and DSC showed that there is the presence of a linear relationship between the BMI and the AHI but also between the AHI and DSC class, confirming the previous analyses.

As the BMI increases by one point, the AHI will increase by 0.18, while, if the patient belongs to the third class, the AHI will decrease by 3.38. The effects of the other co-variants are inconsistent.

Therefore, we can conclude that it is more likely that a patient with an AHI > 5 belongs to DSC II than to DSC III.

In support of this finding, we consider the R-square, a statistical index for assessing the accuracy of the model, which in this case is about 54%.

## 4. Discussion

Several reports show that OSAS affects 5–20% of the adult population; some reports indicate an incidence of up to 26%. OSAS seems to be three times more common in men than in women [8,9].

As reported in the literature, 50% of OSAS patients are underdiagnosed and that represents a major public health problem [8].

From the data that emerged as a result of our statistical analyses, there is a significant percentage (33%) of patients who undergo orthognathic surgery with an AHI > 5 and also a percentage of patients (11%) who are “borderline”. These rates are higher than the universally accepted proportion of the general population with moderate and severe apnea and the limits of the prevalence of OSAS in individuals over 18 years of age and with an AHI > 5 according to the Benjafield study, which is 20.5% [10].

In our study, we considered two classes of dento-skeletal malformations, class II and class III, according to Angle’s classification; in class III, we also considered the presence of maxillary hypoplasia, which is particularly at risk for presenting OSAS. 

The results shows a significantly higher percentage (75%) of DSC II patients with an AHI > 5 than DSC III patients with an AHI > 5, but when also considering maxillary hypoplasia, it turns out to be a risk factor in DSC III patients in the development of obstructive sleep apnea. In fact, although the percentage of the latter with an AHI > 5 is only 16% in our study, 62% turn out to have maxillary hypoplasia. 

Instead, considering the borderline AHI values (>4 but <5), the 14% of patients with DSC II is also higher here compared to 10% of patients in DSC III.

Of the patients with a DSC III malformation, only one out of five also has maxillary hypoplasia, so the data in this case are less significant.

Our results confirm that the pattern most at risk is the one characterized by retruded maxilla and patients with dento-skeletal class II malformations.

This finding was previously documented in the literature; in 2007, George affirmed that patients with a diagnosis of OSAS usually display a class II malocclusion and a retrusive profile [11]. 

As already widely reported in the literature, another significant finding that emerged from the statistical analysis carried out is the correlation between BMI and the AHI which demonstrates how patients with a higher BMI are more at risk of developing apneas and hypopneas during sleep [12].

The statistical analysis we performed shows that after surgery for the correction of a dento-skeletal malformation, only 8% of patients with malformations present an AHI > 5, compared to the 20.5% described in the Italian population [10].

We also described a patient with a worsening of their AHI value; the patient underwent the correction of a DSC III malformation and maxillary hypoplasia.

These data is in agreement with other reports in the literature which indicate that this pattern is the most subject to OSAS [13].

It is therefore possible to hypothesize that there is an increased risk of worsening for patients in this category. 

This could be especially true in cases of surgical planning that involve only modest maxillary advancement with substantial mandibular retraction. These surgical procedures actually reduced the airway volume, which can be inadequate in these patients.

From our study it emerges that in some types of malformations, the risk of treating not only a dysmorphic patient but also a patient with OSA is high. For these patients, pre-surgical planning should involve a comprehensive polysomnography evaluation.

In particular, the surgeon must consider the augmented risk of silent OSAS, especially for patients with dento-skeletal class II and dento-skeletal class III malformations with maxillary hypoplasia.

In patients with a hypo-development of the mandible (DSC II), generally a reduction in sagittal diameter at the oro-pharyngeal and hypo-pharyngeal levels is the reason for stenosis. In these cases, usually mandibular advancement improves these air spaces.

In patients with DSC III malformations and maxillary hypoplasia, the upper third of the upper airway column is hypo-represented while the middle and lower third upper airway column is normo-represented or hypo-represented. The esthetic conformation of the patient might tempt planning to retract the mandible, and this surgical movement would certainly go even further to reduce the volume of the upper airway. In this type of patient, airway study is mandatory before proceeding with any surgical programming. 

From our results and the evidence present in the literature, it is clear that patients suffering from dento-skeletal class II malocclusions are more often, even unconsciously, affected by OSAS. It is useful to remember how, in patients suffering from mandibular retrusion, mandibular advancement devices can represent a valid tool for verifying possible post-surgical improvements of the AHI.

The same cannot be said of patients suffering from maxillary retrusion; this component of malocclusion should always be kept in mind when considering upper airway changes related to scheduled surgical moves [6].

The correlation between OSA and dento-skeletal malformations is well known in the literature. The diagnosis of moderate or severe OSA is a clear indication for the need for orthognathic surgery as reported by several studies [14].

The success rate of orthognathic surgery in moderate and severe OSA is reported to be up to 87.5% [15].

Despite these considerations, there is still little knowledge of how orthognathic surgery can influence cases of silent OSA and how to prevent OSA in patients undergoing orthognathic surgery or in patients at risk for the condition.

Scheduled surgeries not taking into account airway volumes may result in the occurrence or worsening of obstructive airway disease during sleep. This risk is much lower in patients with DSC III malformations from the sagittal overdevelopment of the mandible, and in fact, we did not observe an increase in AHI value after surgery in any patient.

Although this study has some limitations, such as the absence of a CT control for airway study, a non-homogeneous sample, and the non-consideration of the extent of surgical movements, it highlights how the frequency of silent OSAS in the patients we treat for the surgical correction of dento-skeletal malformations is high.

Other limitations of the present study are the retrospective and monocentric design of the study, and that we excluded patients with major BMI fluctuations but changes related to minor BMI were not considered.

It also shows how there is generally an improvement in AHI values, although in rare cases, there could be a worsening which could, for example, lead to a clinical picture of OSAS.

It therefore appears to be clear that polysomnographic study is essential in the pre-operative study of the patient.

## 5. Conclusions

From the evidence that emerged in this study, some conclusions can be highlighted.

In malformed patients who receive orthognathic surgery for the correction of facial dysmorphism, the presence of obstructive sleep apnea is significantly higher than in the general population.

Obstructive sleep apneas are mainly present in patients with a sagittal hypo-development of the mandible (DSC II) and in patients with a sagittal hypo-development of the upper jaw (DSC III with maxillary hypoplasia) where the presence of silent OSA should always be suspected.

The necessity of polysomnographic examination in patients who are about to undergo a process of resolution of facial dysmorphia should be essential as the risk of silent OSA is real and much higher than in the general population.

It is evident from our study that there is a possibility of worsening respiratory function after orthognathic surgery performed without regard for the patient’s AHI value.

When surgically correcting a dento-skeletal malformation, the surgeon must aim not only to a satisfying esthetics, but also to achieve proper stomatognathic and respiratory function. This cannot be achieved without taking polysomnography information into account.

## Data Availability

All data are available from the corresponding authors upon reasonable request.
